# The *Cucurbita ficifolia* Mitochondrial Genome: Repeat-Rich Architecture, Plastid Homology, and Synteny Turnover Across Cucurbitaceae

**DOI:** 10.3390/genes17091103

**Published:** 2026-09-11

**Authors:** Jun Yang, Xinhui Li, Huilin Cheng, Yingfeng Luo, Weifan Wu, Xin Lu, Chengcheng Ling

**Affiliations:** College of Food and Bioengineering, Bengbu University, Bengbu 233000, China; yangj@bbc.edu.cn (J.Y.); lixinhui2026520@163.com (X.L.); 18324790332@163.com (H.C.); luo94770927@163.com (Y.L.); wuweifan0524@163.com (W.W.); luxin@163.com (X.L.)

**Keywords:** *Cucurbita ficifolia*, mitochondrial genome, Cucurbitaceae, dispersed repeats, mitochondrial plastid DNA, RNA editing, phylogenomics, collinearity

## Abstract

**Background:** *Cucurbita ficifolia* (figleaf gourd) is an agriculturally useful cucurbit germplasm and rootstock, but its mitochondrial genome has not been examined in a comparative family-level framework. We aimed to characterize its mitochondrial reference sequence and evaluate coding composition, repetitive DNA, plastid homology, bioinformatic RNA-editing predictions, phylogenetic placement, and genome-scale synteny. **Methods:** PacBio Revio sequencing generated 933,158 high-fidelity (HiFi) reads totaling 14.294 Gb, with a read N50 of 15.6 kb. The 784,544-bp mitochondrial reference (GenBank accession PZ823090) was assembled with Oatk and evaluated using graph topology, competitive read-back, and junction-spanning alignments. Codon usage, repeats, mitochondrial–plastid homologous regions, bioinformatic C-to-U RNA-editing predictions, conserved-gene phylogeny, and whole-mitogenome synteny were analyzed. **Results:** The reference had 43.09% GC content and contained 38 distinct protein-coding genes represented by 42 loci, 26 tRNA types represented by 37 loci, and three rRNAs. Competitive read-back retained 40,971 primary mitochondrial alignments (MAPQ ≥ 20), yielding a mean depth of 547.26× and 100% breadth at ≥100×. The terminal-to-start adjacency was supported by 366 HiFi reads with ≥2-kb anchors on each side. We identified 322 simple-sequence repeats, 115 tandem repeats, and 2374 dispersed repeat pairs. For the largest 392-bp direct repeat, the two native junctions were supported by 268 and 308 reads, whereas the two reciprocal junctions were supported by one and zero reads. Ninety-one significant plastid matches collapsed to 73 mitochondrial loci and covered 74,784 unique mitochondrial bases (9.53%). Deepred-Mt generated 494 bioinformatic C-to-U RNA-editing predictions in 37 genes, and phylogenomics placed *C. ficifolia* as sister to the *C. pepo*–*C. maxima* pair with maximal support despite extensive synteny turnover. **Conclusions:** The *C. ficifolia* mitogenome combines a conserved coding repertoire with abundant short repeats, appreciable plastid homology, and extensive rearrangement. Read evidence supports the submitted terminal-to-start adjacency but does not establish a unique or predominant circular molecule in vivo; likewise, the largest repeat showed no robust reciprocal-junction signal. All RNA-editing sites reported here are bioinformatic predictions rather than transcript–validated events.

## 1. Introduction

Plant mitochondria integrate respiration with broader metabolic and signaling networks, while their genomes follow an evolutionary regime unlike the compact mitochondrial chromosomes of most animals [1,2]. Angiosperm mitogenomes often retain a recognizable set of respiratory and translation genes despite pronounced differences in total length, chromosome number, sequence order, and noncoding content [1,2,3]. Repeat-dependent repair, ectopic recombination, intracellular DNA movement, and lineage-specific sequence turnover all contribute to this contrast between conserved function and labile architecture [3,4,5]. How these processes interact to generate, sort, and maintain alternative chromosome configurations remains unresolved, particularly at repeat-rich loci [1,3,4,5]. Consequently, a circular assembly is best interpreted as a navigable reference path rather than direct evidence that one circular molecule predominates in vivo [4].

Cucurbitaceae provide an unusually informative setting for studying such variation. Early complete sequences showed that the 379-kb watermelon and 983-kb zucchini mitogenomes differ markedly in repeat and plastid-derived content [6], whereas the melon mitochondrial genome approaches 2.7 Mb and includes extensive sequence of nuclear origin [7]. Subsequent studies expanded taxonomic and intraspecific sampling to *Cucumis hystrix* [8], independently assembled bottle gourd (*Lagenaria siceraria*) mitogenomes [9,10], a long-read analysis of 12 cucumber accessions with extensive intraspecific structural variation [11], and *Benincasa hispida* [12]. Together, these studies reveal that genome size, repeat architecture, chromosome representation, and organellar homology can change substantially within one crop family. These contrasts make close-relative comparisons especially informative for separating lineage-specific changes in repeat architecture, intracellular homology, and genome order from broadly conserved coding features.

Figleaf gourd (*C. ficifolia* Bouché), often called black-seed squash, is cultivated as a food plant and used as a rootstock and germplasm source for stress and disease resistance. Population resequencing has documented environmental associations and limited geographic structure among Chinese accessions [13], while population-genomic work on domesticated material indicated a recent bottleneck and restricted gene flow with wild relatives [14]. Complete plastomes have also provided sequence resources and phylogenetic markers for the species [15,16], complementing broader chloroplast-based reconstructions of *Cucurbita* relationships [17]. To our knowledge, no previous study has combined repeat analysis, mitochondrial–plastid homology, bioinformatic RNA-editing prediction, conserved-gene phylogeny, and whole-mitogenome synteny for *C. ficifolia*.

Plant mitogenome research still faces unresolved questions about how repeat size and genomic context influence structural change, how alternative configurations are maintained, and why extensive genome-order turnover coexists with conservation of mitochondrial coding sequences [3,4,5,18,19,20]. Guided by these themes but focused on Cucurbitaceae, the present study characterizes the mitochondrial reference (GenBank accession PZ823090) of *C. ficifolia*. We addressed five questions: (i) how its genome size and gene repertoire are positioned relative to those reported for other cucurbits; (ii) what repeat classes and size distributions characterize its architecture; (iii) what fraction is homologous to the plastome and what those regions can—and cannot—reveal about intracellular transfer; (iv) what bioinformatic C-to-U RNA-editing predictions are generated for the mitochondrial coding genes; and (v) whether conserved-gene phylogeny is accompanied by conservation of genome order.

## 2. Materials and Methods

### 2.1. Sequence Resource, Genome Assembly and Annotation

Plant material of *C. ficifolia* accession CK648 was provided by the Yunnan Academy of Agricultural Sciences, Yunnan, China. Fresh young leaves from healthy plants were collected for DNA extraction. High-quality total genomic DNA was extracted using a DNAsecure Plant Kit (Tiangen Biotech Co., Ltd., Beijing, China) according to the manufacturer’s instructions. DNA concentration, purity, and integrity were evaluated before library preparation. A SMRTbell library was constructed and sequenced on a PacBio Revio single-molecule real-time sequencing platform (Pacific Biosciences, Menlo Park, CA, USA) by Biomarker Technologies Corporation (Beijing, China). Circular consensus sequencing generated 933,158 high-fidelity (HiFi) reads totaling 14,294,090,707 bp (14.294 Gb), with a mean read length of 15,318 bp and a read N50 of 15,600 bp. Bases with predicted quality scores of at least Q20 and Q30 accounted for 97.44% and 93.94% of the dataset, respectively.

The mitochondrial genome was assembled from the PacBio HiFi reads using Oatk (*syncasm* v1.0; Bioconda build 1.0-1) [21]. The *syncasm* module constructed a sparse de Bruijn graph with *k* = 1001, minimum coverage = 30, and 32 threads (*syncasm -k 1001 -c 30 -t 32*). Because total genomic DNA was sequenced, mitochondrial DNA was not physically purified from nuclear or plastid DNA. Instead, separation was performed computationally after graph construction: hmmannot v1.0 identified unitigs containing land-plant mitochondrial-profile hidden Markov model hits, and these unitigs anchored pathfinder v1.0. Pathfinder resolved the mitochondrial path by integrating organelle-gene annotations, graph connectivity, sequence coverage, and repeat relationships; only unitigs assigned to the resolved mitochondrial path were incorporated into the reference. Plastid-homologous sequence embedded within that path was retained because it can represent genuine mitochondrial integration and was evaluated separately against plastome NC_058583.1 (Section 2.4). The final Oatk graph was inspected for connectivity, unresolved branches, and terminal overlap, and it was rendered in Bandage [22] (Supplementary Figure S1). A single 784,544-bp nonredundant path with no ambiguous bases was retained as the circular reference representation; this representation was not assumed to be the unique or predominant physical chromosome in vivo.

For post-assembly validation, HiFi reads were competitively aligned against the final mitochondrial and plastid contigs with minimap2 v2.28-r1209 (*-ax map-hifi --secondary=no --sam-hit-only --MD -t 60*); alignments were processed with samtools v1.19.2/htslib v1.21. Primary mitochondrial alignments with MAPQ ≥ 20 were retained, and per-base depth was calculated from bases with quality ≥10; breadth was summarized at thresholds from 1× to 100×, and depth was plotted in nonoverlapping 5-kb windows (Supplementary Figure S2). To test the terminal-to-start adjacency independently, a 40-kb synthetic reference joined the final 20 kb to the first 20 kb of the mitochondrial sequence. A primary alignment with MAPQ ≥ 20 was counted as junction spanning only when it contained the required aligned sequence on both sides; support was summarized at 1-, 2-, and 5-kb two-sided anchor thresholds (Supplementary Figure S1).

Initial gene annotation was performed using PMGA v1.0 and its curated plant mitochondrial genome database [23]. The annotated mitochondrial genome of *Cucurbita pepo* (GenBank accession NC_014050.1) was used as the primary reference for identifying protein-coding genes and checking gene boundaries [6]. The predicted protein-coding genes were additionally compared with homologous mitochondrial genes from other Cucurbitaceae species to verify gene identity, exon organization, and potential gene losses. Transfer RNA genes were independently predicted using tRNAscan-SE v2.0.11 [24], whereas ribosomal RNA genes were confirmed through BLASTN v2.16.0 searches [25]. Gene boundaries, exon–intron junctions, start and stop codons, and cis- and trans-spliced genes were manually examined and corrected in Apollo v1.11.8 [26]. The final circular genome map was drawn using OGDRAW v1.3.1 [27].

### 2.2. Gene Inventory and Codon–Usage Analysis

The GenBank annotation for PZ823090 was treated as the starting annotation. Protein-coding, tRNA, and rRNA features were counted both as physical loci and as distinct gene types so that duplicated loci did not inflate gene diversity. Multipart coding sequences were reconstructed from annotated joins in strand-aware order. The codon_start qualifier was respected, and all complete codons from the 42 annotated coding features were counted before applying the bioinformatic RNA-editing predictions. Relative synonymous codon usage (RSCU) was calculated as the observed count of a codon divided by the expected count under equal usage of all synonymous codons for the same amino acid. Stop codons were summarized separately. The circular map and codon figure were produced from the audited sequence and feature table using custom Python v 3.14.7 scripts.

### 2.3. Repeat Detection and Read-Based Junction Testing

SSR screening followed the six MISA minimum-copy cutoffs—10, 5, 4, 3, 3, and 3—for motif lengths of one through six nucleotides [28]. Tandem Repeats Finder was run with settings 2 7 7 80 10 50 500 [29]. ROUSFinder then identified direct and inverted (palindromic) homologies ≥ 30 bp [30], which were summarized by orientation and length class.

Pairs of at least 50 bp were retained as candidate repeat-associated junctions. For each of 259 pairs, four reference paths were constructed, giving 1036 paths in total: two represented the assembly-native junctions and two represented reciprocal junctions expected after repeat-mediated exchange. The largest repeat, R1, was then tested directly using all raw whole-genome HiFi reads without preselection against the final mitochondrial assembly. Two native and two reciprocal 12,392-bp synthetic references each comprised the 392-bp repeat and 6-kb unique flanks on both sides. Reads were aligned with minimap2 v2.28-r1209 using *-x map-hifi -c --cs=long --secondary=yes -N 20 -p 0.5 -t 60*. A supporting alignment was required to be continuous across the repeat and both flanks, have identity ≥98% and MAPQ ≥ 20, and include ≥2000 bp of unique sequence on each side; reads satisfying more than one path were classified as ambiguous and excluded. Because this stringent test was applied only to R1, the remaining 258 candidate pairs were not assigned read-based support classes (Supplementary Figure S3).

### 2.4. Mitochondrial–Plastid Homology

The mitochondrial reference was compared with plastome NC_058583.1 using BLASTN, and hits with E-values ≤ 1 × 10^−5^ were retained [25]. Exact duplicate mitochondrial coordinate pairs among BLAST v2.16.0 high-scoring segment pairs (HSPs) were collapsed to estimate the number of distinct homologous loci. Interval-union coverage was calculated separately for mitochondrial and plastid coordinates so that each reference position was counted once despite overlapping HSPs or alignments to duplicated plastid inverted-repeat sequences. Alignment-length-weighted identity was calculated across retained HSPs. Homologous segments were displayed with Circos [31]. Because similarity alone does not establish transfer direction or timing, these regions are described as mitochondrial–plastid homologous segments or MTPTs.

### 2.5. Bioinformatic Prediction of RNA-Editing Sites

Potential C-to-U RNA-editing sites were predicted bioinformatically in mitochondrial coding sequences with Deepred-Mt [32]. Only predictions with posterior probability > 0.90 were retained. Counts were summarized by gene and predicted amino-acid replacement. Because no matched mitochondrial transcriptome was analyzed, all reported sites—including those predicted to complete stop codons—are bioinformatic predictions rather than observed editing events.

### 2.6. Phylogenomic Analysis

Twenty-four mitochondrial protein-coding genes shared across the taxon set were aligned individually with MAFFT v7.520 [33] and concatenated into a 23,994-bp nucleotide matrix. A maximum-likelihood tree was inferred with IQ-TREE v2.2.3 [34] using the command options *-m MFP --alrt 1000 -B 1000*; ModelFinder selected GTR+F+R2 under the Bayesian information criterion [35]. Branch support was evaluated with 1000 SH-aLRT replicates and 1000 ultrafast bootstrap replicates. *Glycine max* and *Phaseolus vulgaris* were used as outgroups. The ingroup included publicly available Cucurbitaceae mitogenomes sharing the 24-gene set, with dense sampling of *Cucurbita*, whereas the two Fabales species provided an external root. The topology was formatted in iTOL v7.6 [36].

### 2.7. Whole-Mitogenome Collinearity

Adjacent genomes in the ordered comparison (*C. maxima* → *C. ficifolia* → *C. pepo* → *L. siceraria* → *C. lanatus* → *C. sativus*) were aligned with minimap2 v2.30-r1287 using -x asm10 -c --eqx --secondary=yes -N 1000 -p 0.5 [37]. The design prioritized the two available congeneric references, *C. maxima* and *C. pepo*, and then sampled four additional Cucurbitaceae lineages with public annotated mitogenomes, contrasting genome sizes, and different structural representations; accessions and principal parameters are listed in Supplementary Table S8. Alignments shorter than 1000 bp or with identity below 90% were removed. Forward and reverse blocks were counted separately, and the sum of alignment lengths, longest retained block, and alignment-length-weighted identity were calculated for each adjacent pair. These sums describe retained alignments and are not interpreted as nonoverlapping genome coverage.

## 3. Results

### 3.1. Genome Organization, Assembly Validation, and Gene Content

**Figure 1:**
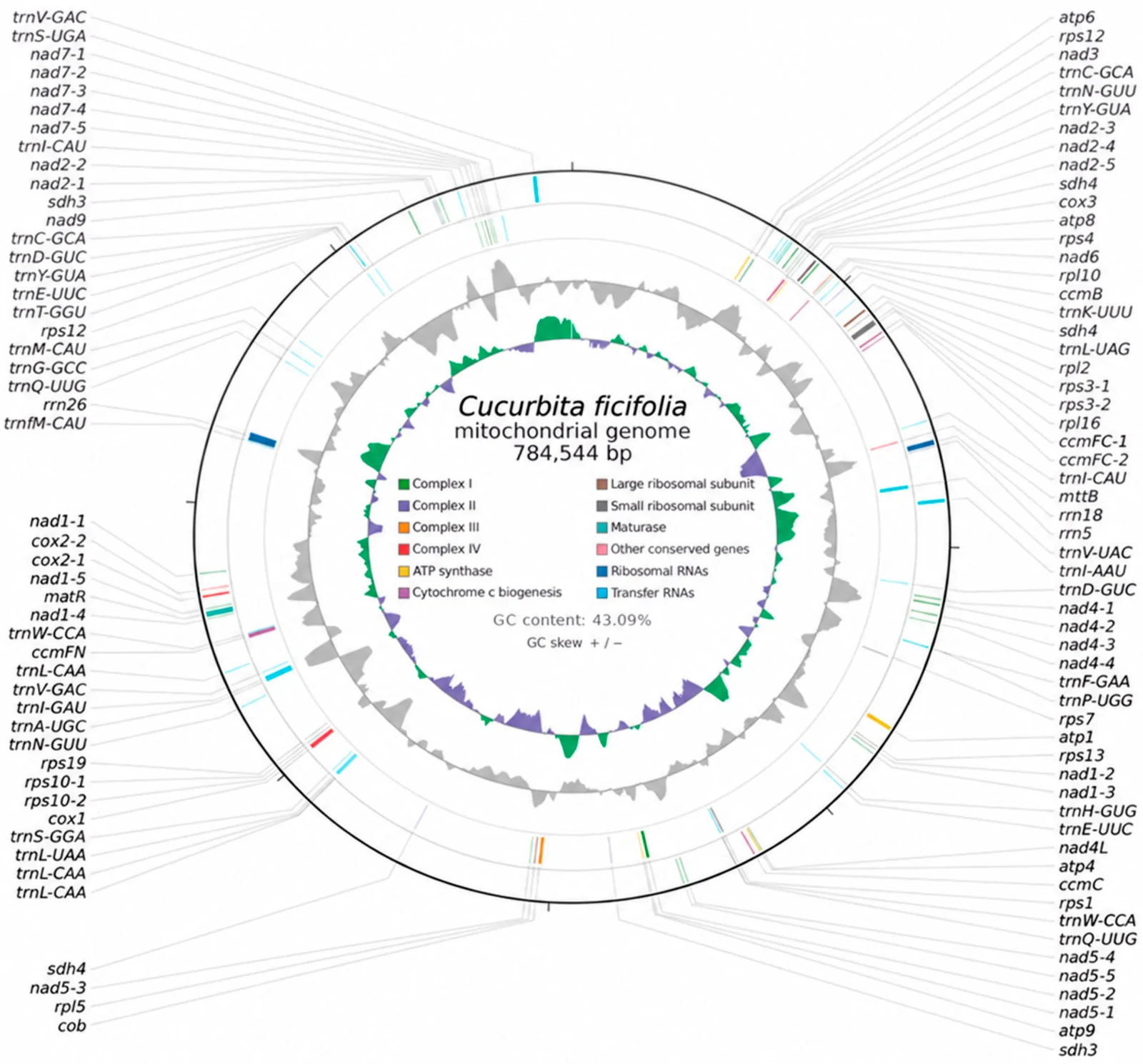
Circular map of the *Cucurbita ficifolia* PB_CK648 mitochondrial reference (GenBank PZ823090). Genes outside and inside the circle are transcribed in opposite directions. Colored bars identify functional categories, and the inner tracks show GC content and GC skew. Exons of multipart genes are numbered. The center reports the 784,544-bp sequence length. The circle is a reference representation and not a model of the predominant physical chromosome in vivo.

The final Oatk graph contained a single 784,977-bp segment, u4173, with a 433-bp terminal self-link; removing the duplicated terminal overlap produced the 784,544-bp nonredundant reference (43.09% GC) with zero ambiguous bases (Table 1 and Table 2; Figure 1; Supplementary Figure S1). Its 82 annotated gene loci represented 67 distinct gene types: 42 protein-coding loci corresponding to 38 distinct PCGs, 37 tRNA loci corresponding to 26 anticodon-defined tRNA types, and three rRNA loci (*rrn5*, *rrn18*, and *rrn26*) (Table 1). The duplicated coding genes were *rps12* (two loci), *sdh3* (two loci), and *sdh4* (three loci).

The *C. ficifolia* PB_CK648 mitochondrial reference (GenBank PZ823090) was assembled from 933,158 HiFi reads totaling 14.294 Gb (read N50, 15,600 bp) (Table 2). Competitive read-back retained 40,971 primary mitochondrial alignments with MAPQ ≥20 (Table 2). After excluding bases with quality <10, the mean and median depths were 547.26× and 507×, respectively (range, 240–3285×), and every reference position was covered at ≥100× (Table 2; Supplementary Figure S2). The terminal-to-start junction was spanned by 483, 366, and 159 primary reads with ≥1-, ≥2-, and ≥5-kb anchors on each side, respectively (Table 2; Supplementary Figure S1). These data support continuous coverage and the terminal-to-start adjacency used in the submitted reference, but they do not identify the unique or predominant genome-scale conformation in vivo.

The coding complement included the 24 widely conserved angiosperm mitochondrial genes: *atp1*, *atp4*, *atp6*, *atp8*, *atp9*; *nad1*–*nad7*, *nad4L*, and *nad9*; *ccmB*, *ccmC*, *ccmFC*, and *ccmFN*; *cob*; *cox1*–*cox3*; *matR*; and *mttB*. Four large-subunit ribosomal protein genes (*rpl2*, *rpl5*, *rpl10*, and *rpl16*), eight small-subunit ribosomal protein gene types (*rps1*, *rps3*, *rps4*, *rps7*, *rps10*, *rps12*, *rps13*, and *rps19*), and *sdh3* and *sdh4* completed the variable coding set.

The graph and read-back metrics provide strong sequence-level support for the submitted reference and its terminal-to-start adjacency (Supplementary Figures S1 and S2), but they do not establish that a single 784,544-bp circular molecule is the unique or predominant configuration in vivo.

### 3.2. Codon-Usage Pattern

**Figure 2:**
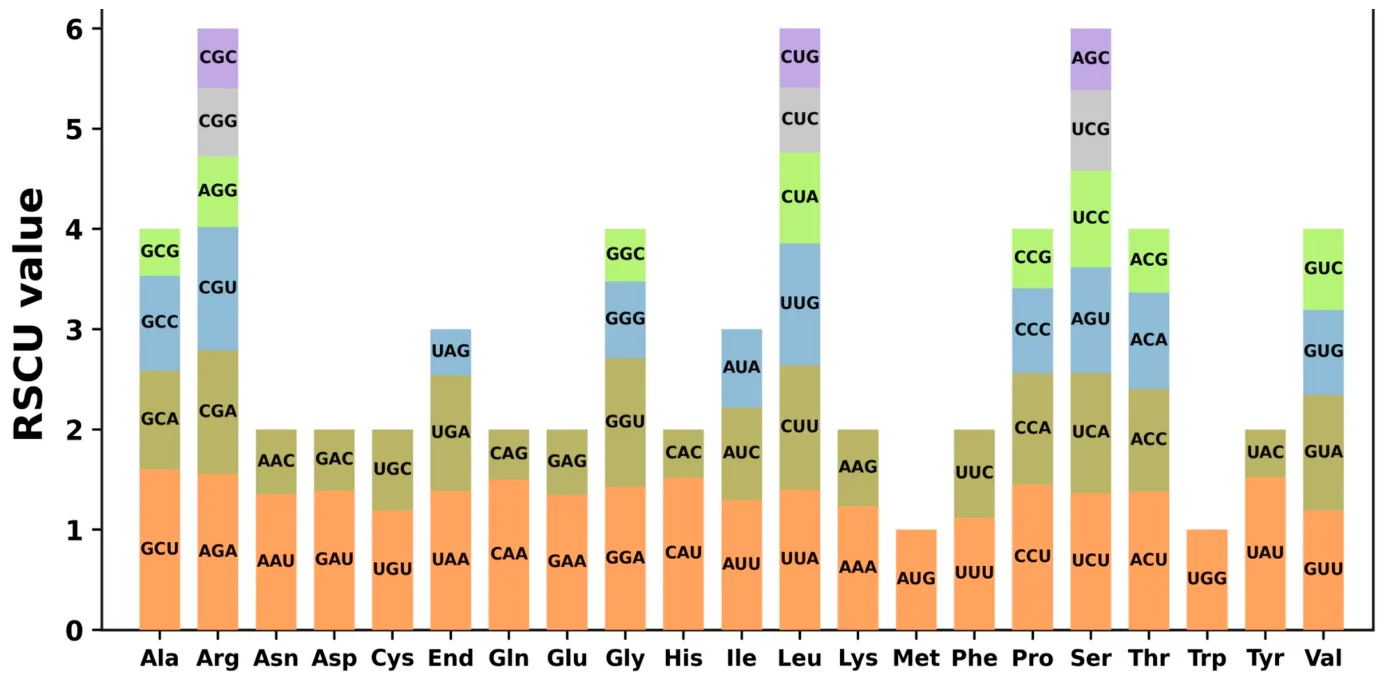
Relative synonymous codon usage (RSCU) in the *Cucurbita ficifolia* mitochondrial coding set, with codons in RNA notation. Segment values above 1 indicate preferential use; bar height indicates family degeneracy rather than bias strength.

The 42 coding features contributed 10,954 complete codons, including 10,915 sense codons and 39 terminal stop codons. No ambiguous codons or internal stops were detected. For each of the 18 multi-codon amino acids, the preferred codon ended in A or U (Figure 2). The strongest biases were observed for GCU (Ala; RSCU 1.60), AGA (Arg; 1.55), UAU (Tyr; 1.52), CAU (His; 1.52), CAA (Gln; 1.50), CCU (Pro; 1.45), GGA (Gly; 1.42), and UUA (Leu; 1.40). Among stop codons, UAA occurred 18 times (RSCU 1.38), UGA 15 times (1.15), and UAG six times (0.46).

### 3.3. Repeat Landscape and Read-Based Evaluation of the Largest Repeat

**Figure 3:**
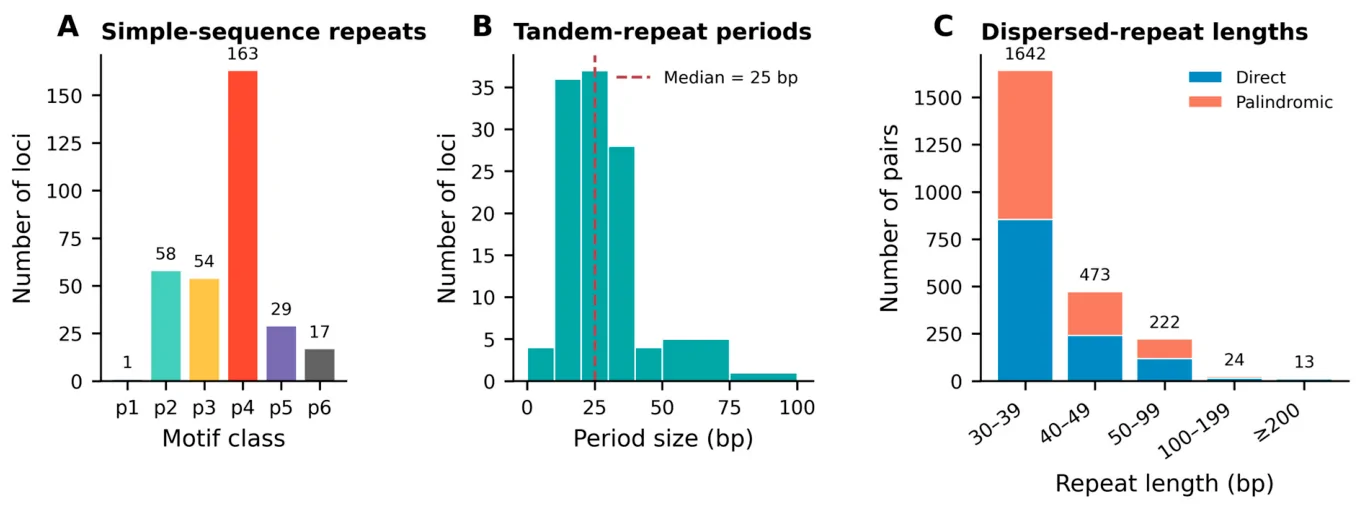
Repeat landscape of the *Cucurbita ficifolia* mitochondrial reference. (**A**) SSR counts by motif class (p1–p6). (**B**) Distribution of tandem-repeat period sizes; the dashed line indicates the median. (**C**) Dispersed repeat pairs by length and orientation. Direct and palindromic pairs are shown separately. Counts describe sequence pairs and not recombination events.

The mitochondrial reference contained 322 SSRs, 115 tandem repeats, and 2374 dispersed repeat pairs (Table S3; Figure 3). Tetranucleotide SSRs were the dominant class (163; 50.62%), followed by dinucleotide (58), trinucleotide (54), pentanucleotide (29), hexanucleotide (17), and mononucleotide (1) loci. SSR sizes ranged from 10 to 30 bp. Tandem-repeat periods ranged from 5 to 93 bp (median 25 bp), with inferred copy numbers of 1.9–13.6. The dominance of short motifs identifies an abundant pool of potential microhomology, but repeat counts alone do not demonstrate structural exchange.

The dispersed-repeat catalog comprised 1237 forward and 1137 palindromic pairs. Most were short: 1642 pairs were 30–39 bp and 473 were 40–49 bp, together accounting for 89.09% of the catalog. Only 259 pairs were at least 50 bp and 37 were at least 100 bp. The largest repeat, R1, was a 392-bp direct pair at positions 92,091–92,482 and 680,968–681,359. Under the prespecified MAPQ, identity, and ≥2-kb anchor criteria, the two native R1 junctions were supported by 268 and 308 uniquely assigned HiFi reads, respectively (Supplementary Figure S3). Only one read supported candidate reciprocal junction A, and none supported reciprocal junction B. This isolated asymmetric signal was treated as a candidate recombinant-like adjacency rather than robust evidence of reciprocal recombination or a quantitative estimate of molecular stoichiometry. The other 258 repeat pairs were not subjected to the same raw-read test.

### 3.4. Mitochondrial–Plastid Homologous Segments

**Figure 4:**
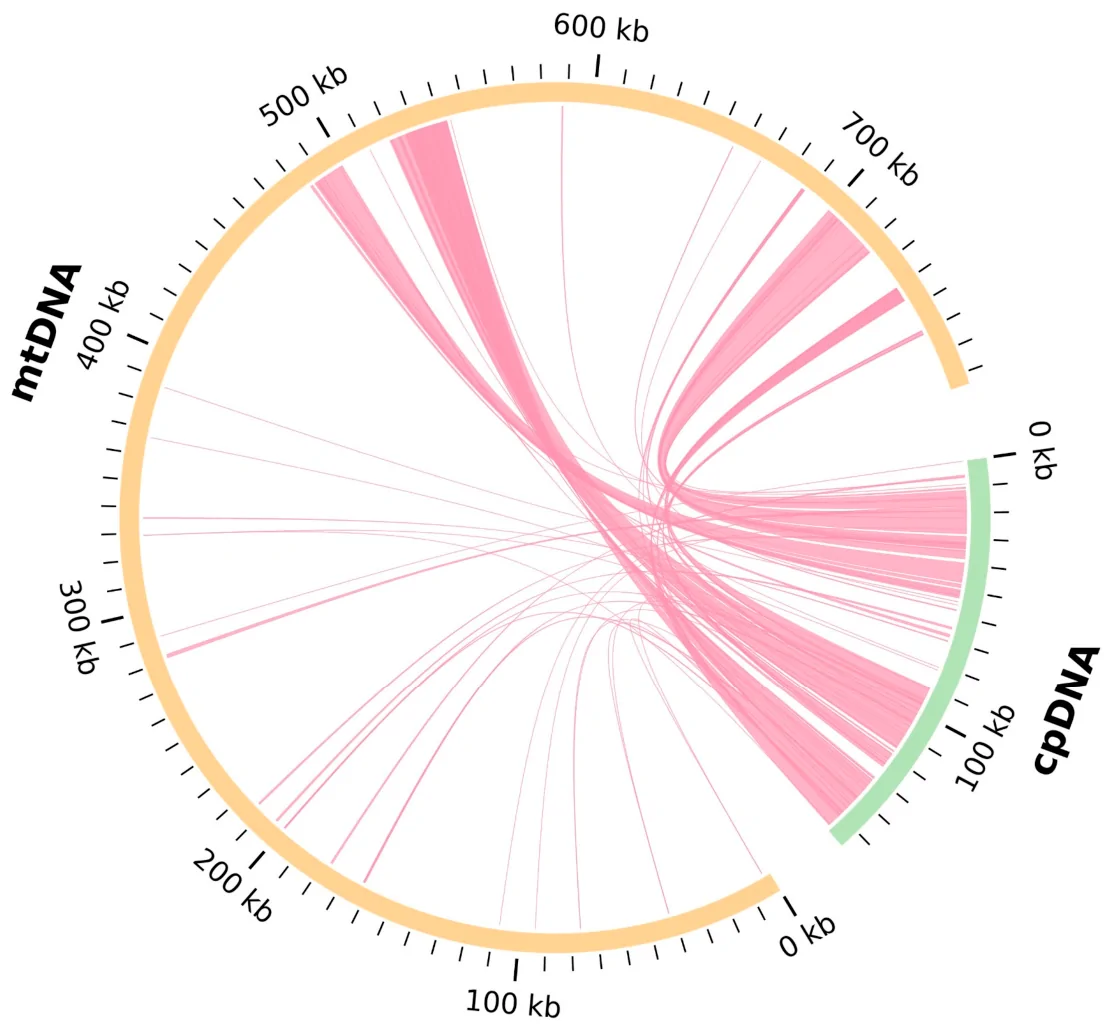
Homologous segments between the *Cucurbita ficifolia* mitochondrial reference (mtDNA) and plastome NC_058583.1 (cpDNA). Ribbons connect significant BLASTN matches. Scale marks are in kilobases. Link density reflects overlapping and repeated HSPs and is not a count of independent transfer events.

Comparison with the *C. ficifolia* plastome NC_058583.1 recovered 91 significant HSPs. After exact duplicate mitochondrial coordinates were collapsed, these matches represented 73 mitochondrial loci (Figure 4; Table S4). The raw sum of HSP lengths was 102,474 bp, whereas interval union gave 74,784 unique mitochondrial bases, equivalent to 9.53% of the mitogenome. On the plastome, homologous intervals covered 96,414 bp (61.20%); this larger fraction includes sequences duplicated in the plastid inverted repeats and therefore should not be interpreted as a single transfer event.

Individual HSPs ranged from 38 to 9003 bp and from 74.77% to 100% identity; alignment-length-weighted identity was 94.44%. The longest HSP was 9003 bp (96.66% identity) and spanned homologs annotated as partial *rpoC2*, complete *rpoC1*, and complete *rpoB*. Other segments contained complete or partial plastid coding and tRNA annotations. At the sequence level, these loci expand plastid–homologous space and may provide substrates for gene conversion or rearrangement, but their expression, function, transfer direction, and timing cannot be inferred from BLAST similarity alone.

### 3.5. Bioinformatic RNA–Editing Predictions

**Figure 5:**
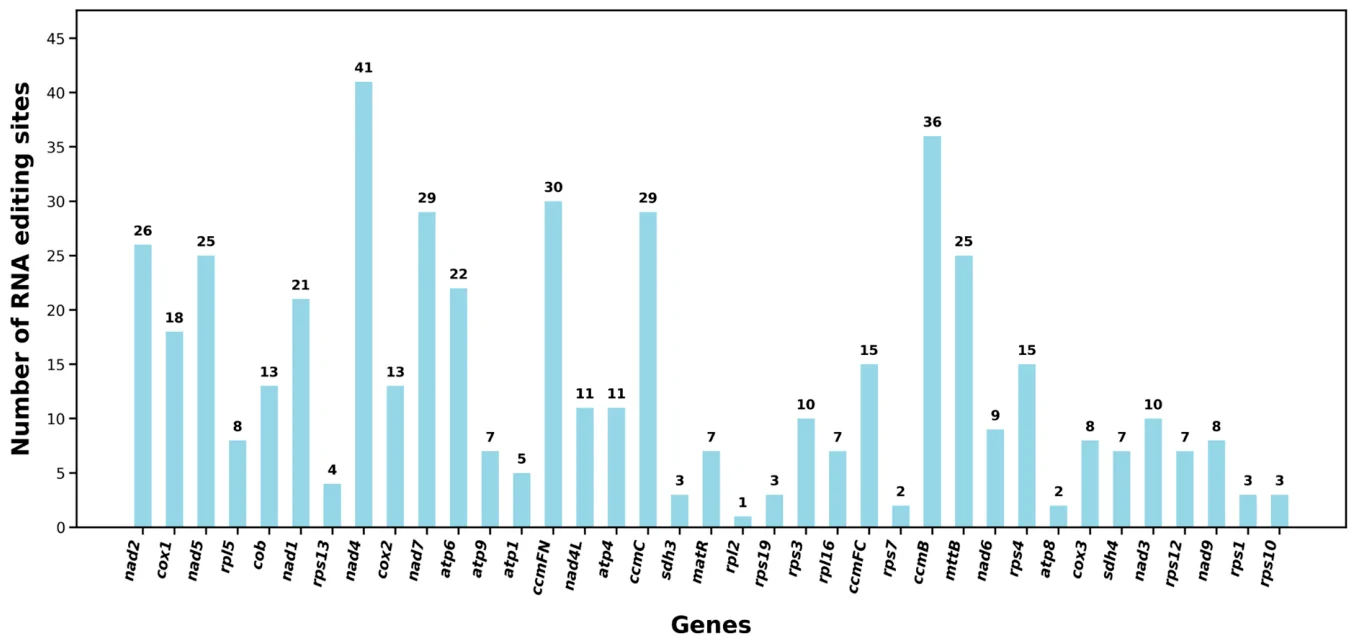
Bioinformatically predicted C–to–U RNA–editing sites in 37 mitochondrial protein–coding genes of *Cucurbita ficifolia*. Bars show the number of predictions retained at Deepred–Mt posterior probability > 0.90, and values are printed above bars. These values are bioinformatic predictions, not observed or transcript–validated editing events.

Deepred-Mt generated 494 high-probability bioinformatic C-to-U RNA-editing predictions in 37 coding genes (Figure 5). Prediction probabilities ranged from 0.903 to 1.000 (mean 0.988). *nad4* had the largest number of predictions (41), followed by *ccmB* (36), *ccmFN* (30), *ccmC* and *nad7* (29 each), *nad2* (26), and *mttB* and *nad5* (25 each). The most frequent predicted amino-acid replacements were Ser→Leu (111 predictions), Pro→Leu (108), Ser→Phe (62), Pro→Ser (46), Arg→Cys (38), and Arg→Trp (35). Within the prediction set, Ser→Leu and Pro→Leu substitutions together accounted for 44.33% of candidates; the corresponding protein-level changes remain hypotheses derived from bioinformatic predictions rather than observed transcript- or protein-level events. Seven Phe→Phe and six Ser→Ser predictions were synonymous. Because no transcript reads were analyzed, these distributions describe bioinformatic model output rather than observed editing frequencies.

### 3.6. Phylogenetic Placement Within Cucurbitaceae

**Figure 6:**
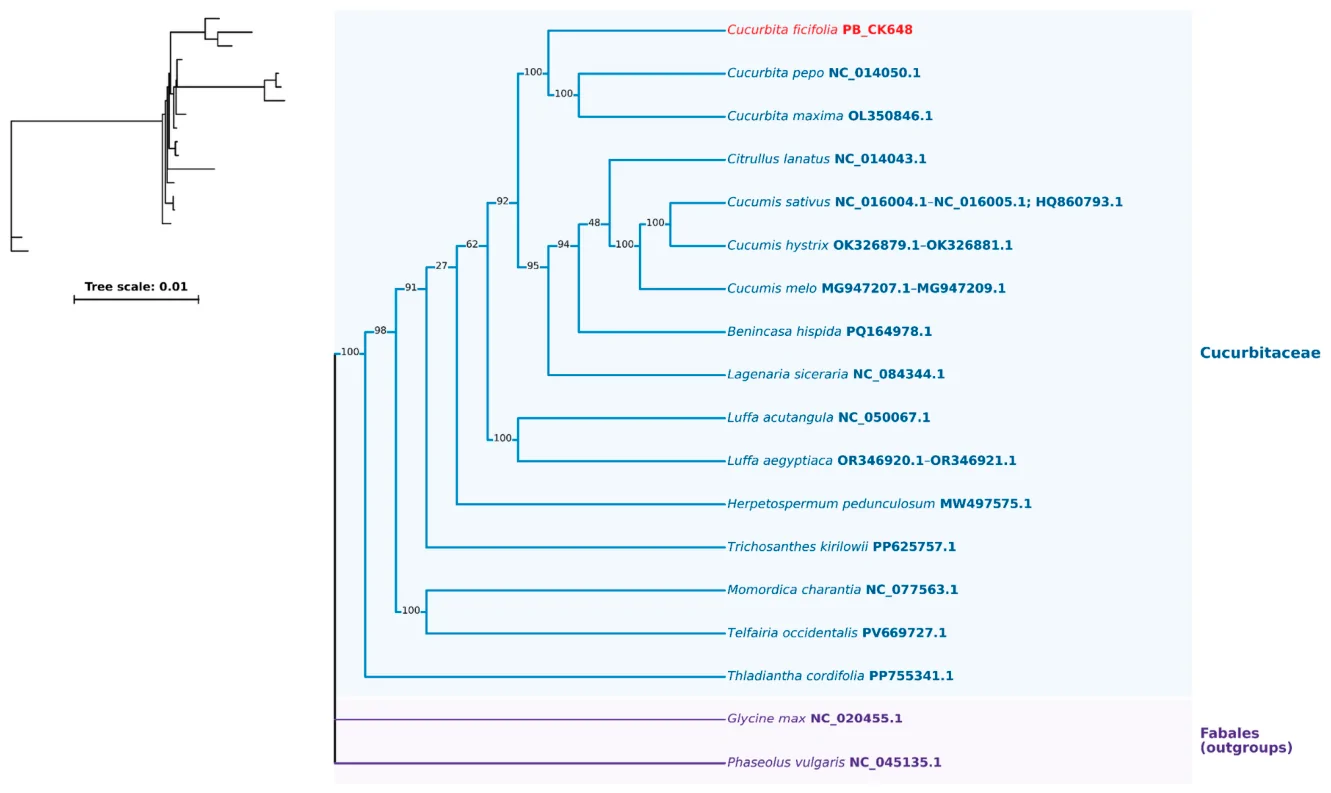
Maximum-likelihood phylogeny based on 24 conserved mitochondrial protein-coding genes. The tree was inferred under GTR+F+R2 with 1000 SH-aLRT replicates and 1000 ultrafast bootstrap replicates; node values show ultrafast bootstrap percentages. *Cucurbita ficifolia* PB_CK648 (PZ823090) is highlighted in red; *Glycine max* and *Phaseolus vulgaris* are outgroups. The inset shows branch lengths, with a scale of 0.01 substitutions per site.

The maximum-likelihood analysis of 24 conserved mitochondrial PCGs recovered a strongly supported Cucurbitaceae clade (Figure 6). *Cucurbita pepo* and *C. maxima* formed a sister pair with 100% ultrafast bootstrap support, and *C. ficifolia* was sister to that pair with 100% support. The remaining topology separated *Citrullus*, *Cucumis*, *Benincasa*, *Lagenaria*, *Luffa*, *Herpetospermum*, *Trichosanthes*, *Momordica*, *Telfairia*, and *Thladiantha* lineages, while the two Fabales accessions formed the outgroup. Thus, the conserved-gene analysis places *C. ficifolia* PB_CK648 (PZ823090) as sister to the *C. pepo*–*C. maxima* pair, while making no assumption that whole-genome order is conserved.

### 3.7. Extensive Synteny Turnover

**Figure 7:**
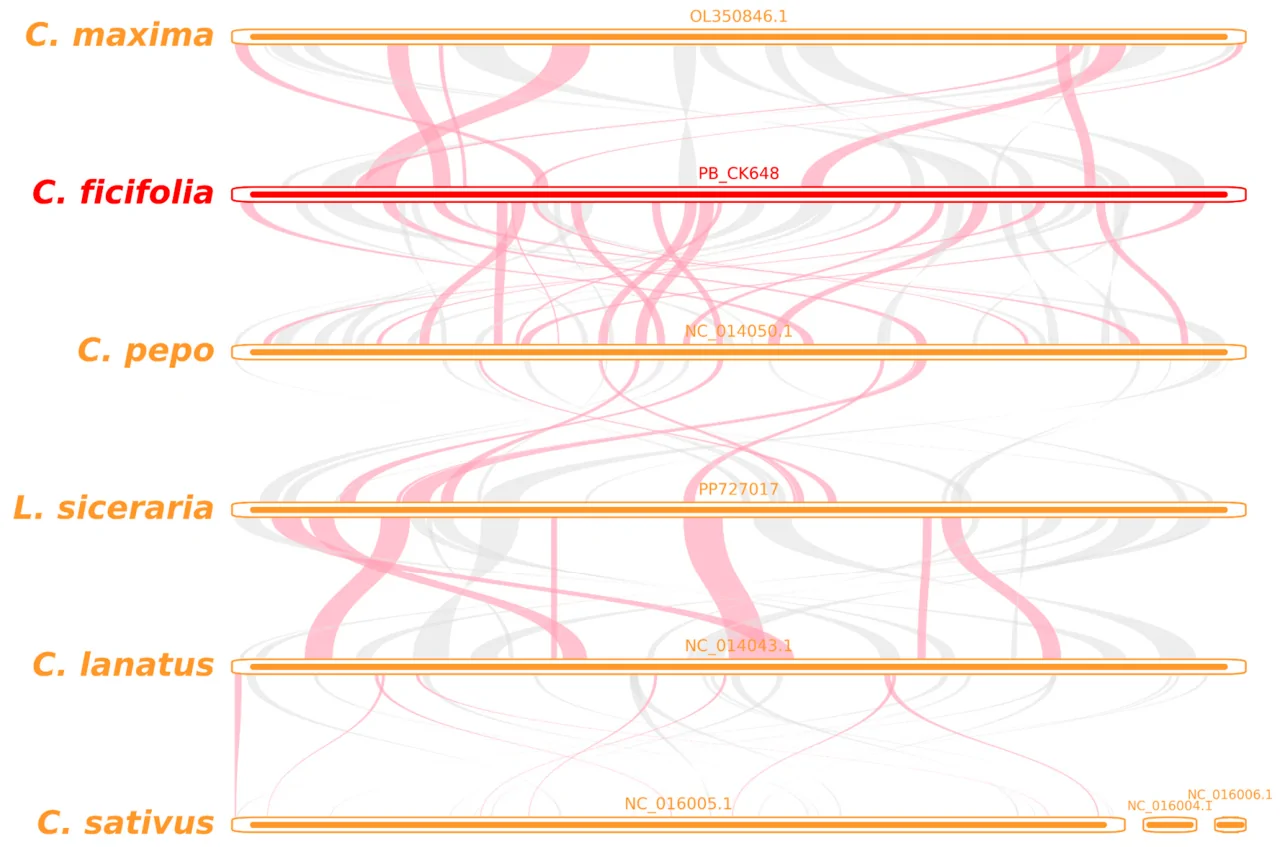
Whole-mitogenome collinearity across six Cucurbitaceae species. Pink links indicate forward-orientation alignments, gray links indicate reverse-orientation alignments, and the red track marks the focal *Cucurbita ficifolia* reference. Only alignments ≥ 1000 bp and ≥90% identity are shown. Forward and reverse blocks describe homologous-sequence orientation; graphical ribbon crossings are not treated as independent evidence of rearrangement or ongoing recombination.

After filtering, the five adjacent genome comparisons retained 131 collinear blocks: 52 in forward and 79 in reverse orientation (Figure 7; Table S7). The *C. maxima*–*C. ficifolia* comparison contained 21 blocks totaling 235,983 aligned bases, including 13 reverse blocks; its longest block was 28,506 bp and weighted identity was 94.15%. The *C. ficifolia*–*C. pepo* comparison retained 42 blocks totaling 313,464 bases, evenly divided between orientations, with a longest block of 22,065 bp and 92.05% weighted identity.

The remaining comparisons retained fewer aligned bases: 86,719 bp for *C. pepo*–*L. siceraria*, 146,804 bp for *L. siceraria*-*C. lanatus*, and 47,761 bp for *C. lanatus*-*C. sativus*. Reverse-orientation blocks outnumbered forward blocks in four of the five comparisons and were equal only for *C. ficifolia*-*C. pepo*. Together, these alignments show that conserved mitochondrial genes can provide well-supported phylogenetic signal while homologous-block orientation and intergenic context turn over extensively; because blocks can overlap and do not establish ancestral states, they do not identify specific rearrangement events.

## 4. Discussion

### 4.1. Genome Size and Conserved Coding Capacity in Cucurbita

At 784,544 bp, the *C. ficifolia* reference is intermediate in size between *C. maxima* OL350846.1 (640,814 bp) and *C. pepo* NC_014050.1 (982,833 bp). It is larger than the bottle-gourd (357,496 bp) and watermelon (379,236 bp) mitogenomes but smaller than the approximately 1.5–1.6-Mb long-read cucumber assemblies [6,10,11]. The newly reported 431,446-bp *Benincasa* assembly further broadens the family-level range [12]. This placement reinforces a central Cucurbitaceae pattern: large differences in mitochondrial sequence space do not require proportional changes in coding capacity. *Cucurbita ficifolia* retains 38 distinct PCGs, a number comparable to those reported for *C. maxima*, *C. hystrix*, cucumber, and *Benincasa* [8,11,12]. The variation therefore resides mainly in duplicated, imported, and lineage-specific noncoding sequence rather than wholesale gain of respiratory genes. Within *Cucurbita*, the 342,019-bp span between the *C. maxima* and *C. pepo* references, with *C. ficifolia* intermediate, illustrates that genome-size evolution can be partly decoupled from the conserved coding repertoire.

Gene copy number nonetheless provides useful structural clues. The extra *rps12*, *sdh3*, and *sdh4* loci in *C. ficifolia* PB_CK648 distinguish physical loci from unique gene types, while the presence of *rpl10* contrasts with its absence in bottle gourd [10]. Across cucurbits, differential retention of ribosomal-protein and tRNA genes has been repeatedly reported [8,10,11,12]. These differences should be interpreted jointly with evidence for nuclear substitution and plastid-derived tRNA acquisition rather than as direct measures of functional gene dosage.

### 4.2. Abundant Short Repeats Indicate Structural Potential, Not Demonstrated Recombination

*C. ficifolia* PB_CK648 contains many dispersed matches, but 89.09% are shorter than 50 bp and the largest is only 392 bp. The predominance of short repeats could reflect repair-associated sequence duplication, persistence of microhomologies in weakly constrained intergenic regions, or lineage-specific gain and loss, although the present data cannot distinguish among these mechanisms [3,38,39]. This profile differs from cucurbit mitogenomes carrying kilobase-scale repeats: the 2026 *Benincasa* assembly contains five dispersed repeats longer than 1 kb, including a 7887-bp repeat [12], and bottle-gourd work used long reads and PCR to validate repeat-associated configurations [9]. Short repeats can participate in repair and rare ectopic exchanges, but repeat length and copy number alone do not establish activity; plant mitochondrial recombination depends on homology, genomic context, surveillance pathways, and stoichiometric selection [38,39].

This distinction is important for comparison with studies that directly quantified or experimentally confirmed alternative junctions [9,18,40,41,42]. For R1, the 268 and 308 reads assigned to the two native junctions greatly exceeded the one and zero reads assigned to the two reciprocal junctions under the same stringent criteria (Supplementary Figure S3). The lack of reciprocal support means that the single candidate read cannot be treated as robust evidence of a stable alternative configuration, and its ratio to native-junction reads is not a recombination-frequency or molecular-stoichiometry estimate. These data show overwhelming support for the native R1 junctions in the sampled reads while leaving open the possibility of very-low-abundance molecules that would require independent validation. Comparable raw-read testing across the other 258 repeat pairs will be required before genome-wide statements about repeat activity can be made.

### 4.3. Mitochondrial–Plastid Homology Is High but Comparable with Recent Cucurbit Reports

Unique mitochondrial coverage by plastid-homologous sequence was 9.53%. This estimate is higher than the 6.50% reported for one bottle-gourd assembly [9] but lower than the approximately 11.35% reported for bottle gourd accession PP727017 [10] and the 11.6% reported for *Benincasa* [12]. The spread among closely related cucurbits likely reflects both biological turnover and analytical choices, including minimum alignment length, treatment of overlapping HSPs, plastid inverted repeats, and whether raw alignment lengths or nonredundant interval unions are reported. Using the union of mitochondrial intervals prevents double-counting of overlapping HSPs and therefore yields a conservative coverage estimate; this is why the summed HSP length (102,474 bp) exceeds the nonredundant mitochondrial coverage (74,784 bp).

The 9-kb *rpoC2*–*rpoC1*–*rpoB* homologous segment is consistent with the retention of large plastid-derived tracts documented in other cucurbit mitogenomes [6,10,12]. Nevertheless, sequence similarity does not uniquely resolve direction: plastid-like segments can enter mitochondria directly, arrive through mitochondrial introgression, or persist as ancient homologs whose subsequent histories differ [43]. Accordingly, the chord diagram is evidence of organellar homology, not a map of 91 independent recent transfer events. Several homologous segments contain complete plastid tRNA annotations, raising the possibility of preferential retention, whereas fragmented protein-coding regions may be nonfunctional. Plastid-derived tracts may also expand intergenic space or provide substrates for ectopic pairing and gene conversion; however, without mitochondrial transcript or proteomic evidence, no identified MTPT is claimed to be expressed, functional, or adaptive.

Plant chloroplast genomes are typically more structurally conserved than mitogenomes, but their repeated elements are not invariant: simple sequence repeats (SSRs), short dispersed repeats, and contraction or expansion at inverted-repeat/single-copy boundaries contribute to plastome length and sequence variation [15,16]. In *C. ficifolia*, plastome resequencing identified 64 mainly A/T-rich SSRs, 296 dispersed repeats concentrated in the 16–20-bp class, 204 single-nucleotide polymorphisms, and 57 indels across 160 individuals; most polymorphism occurred in single-copy and noncoding regions, whereas the inverted-repeat regions were comparatively conserved [16]. This plastome-side variation is relevant to MTPT comparisons because the donor sequence pool can differ among accessions; matched plastome–mitogenome sampling across *Cucurbita* would help distinguish donor-side variation from differential mitochondrial retention and ancestral homology [16,43].

### 4.4. Codon Preference and Bioinformatic Editing Predictions Are Consistent with a Cucurbit Pattern

The A/U-ending preference across all 18 multi-codon amino acids agrees with recent bottle-gourd and *Benincasa* analyses [10,12]. *C. ficifolia* PB_CK648 also yielded 494 bioinformatic RNA-editing predictions, close to the 497 predictions reported for bottle gourd, 501 for *C. hystrix*, and 488 for *Benincasa* [8,10,12]. The concentration of candidates in *nad4*, cytochrome *c* maturation genes, and other respiratory loci also resembles these cucurbit prediction profiles. If experimentally confirmed and translated as predicted, the two most frequent inferred replacements, Ser→Leu and Pro→Leu, would increase leucine content; only the former would represent a hydrophilic-to-hydrophobic change. This bioinformatic pattern is consistent with previously reported profiles of plant mitochondrial C-to-U editing [32,44], but it is not evidence of editing in the analyzed transcripts.

Similarity in predicted site counts across species supports comparison at the bioinformatic-prediction level; however, differences among algorithms and thresholds limit direct numerical comparison. Deepred-Mt scores sequence contexts, whereas actual editing can vary by tissue, developmental stage, nuclear genotype, and transcript abundance. Future matched RNA-seq with adequate mitochondrial transcript coverage, supplemented by targeted RT-PCR and Sanger sequencing, could test site occupancy, editing levels, and tissue-specific variation.

### 4.5. Conserved Gene Phylogeny Coexists with Rapid Genome-Order Turnover

The 24-gene tree places *C. ficifolia* as sister to the *C. pepo*–*C. maxima* pair with maximal support, consistent with its assignment to *Cucurbita* and with plastid-based evidence that distinguishes *C. ficifolia* from the principal domesticated lineages while retaining genus-level affinity [15,16,17]. By contrast, whole-genome alignments recovered fragmented homologous blocks in both orientations; graphical ribbon crossings were not treated as independent evidence of rearrangement. Even the two comparisons involving *C. ficifolia* contained 34 reverse-orientation blocks in total, illustrating how conserved mitochondrial coding sequences can retain phylogenetic information while flanking sequence order is repeatedly rearranged.

This pattern is also visible elsewhere in Cucurbitaceae. Using long-read data, You et al. [11] documented intraspecific structural variation and reticulate graph architectures across 12 cucumber accessions; *Benincasa* shares extensive but reordered blocks with other family members [12], and bottle-gourd comparisons reveal a mixture of conserved and inverted regions [10]. The *C. ficifolia* PB_CK648 data therefore fit a family-level pattern in which conserved mitochondrial coding sequences evolve more conservatively than genome architecture. Because collinear blocks were generated from pairwise alignments and include overlaps, they should be read as evidence of homology and orientation, not as a complete reconstruction of ancestral rearrangement events. Repeat-mediated homologous exchange, double-strand-break repair using short homologies, and lineage-specific gain or loss of foreign and intergenic sequence are plausible contributors to the observed turnover, but block orientation alone cannot identify the causal mechanism or polarize individual events [3,38,39,40,41,42]. Within *Cucurbita*, genome size does not mirror the conserved-gene topology: *C. ficifolia* is intermediate in size yet shares a fragmented mosaic of forward- and reverse-orientation blocks with both congeners, a pattern consistent with lineage-specific turnover of noncoding sequence after divergence. Repeat-associated alternative or multichromosomal representations have likewise been reported for *Scutellaria tsinyunensis* and *Salvia miltiorrhiza* [45,46]. Studies of *Fragaria*, *Medicago*, *Agrostis*, *Paphiopedilum*, *Hevea*, and *Camellia* further document lineage-specific variation in mitochondrial genome organization, repeat composition, and intergenomic sequence transfer [47,48,49,50,51,52]. Recent *Camellia* and *Medicago* studies extend this pattern to multi-branch or chromosomal assemblies and to variation in repetitive sequences and MTPT content [53,54,55].

### 4.6. Limitations and Priorities for Validation

This study has three principal structural and comparative limitations. First, the single-segment graph, continuous read depth, and terminal-junction-spanning reads validate the submitted reference adjacency, but they do not establish the full population of mitochondrial molecular conformations or demonstrate that a 784,544-bp master circle is unique or predominant in vivo [4]. Second, stringent raw-read testing was completed only for the largest repeat, whereas the other 258 ≥50-bp repeat pairs remain untested at the same level. Third, the phylogenetic and collinearity analyses rely on heterogeneous public assemblies, so sequencing technology, assembly strategy, and chromosome representation may contribute to apparent structural disparity. The RNA-editing analysis is explicitly limited to bioinformatic predictions and is not interpreted as evidence of observed transcript editing [32]. Accordingly, the graph, coverage, and R1 results narrow—but do not remove—the structural inference boundary (Supplementary Figures S1–S3).

Future work should test terminal and repeat-associated junctions by PCR or droplet digital PCR in independent biological samples and use additional long reads to quantify low-abundance configurations without assuming a master-circle topology [9]. Equivalent raw-read analyses of the remaining repeat pairs would determine whether R1 is representative of the broader repeat catalog. Future matched mitochondrial RNA-seq, followed by targeted RT-PCR and Sanger sequencing, could test the bioinformatic editing predictions and assess tissue-specific editing levels. Finally, standardized reassembly and graph-aware comparison of additional *Cucurbita* accessions would help distinguish biological structural turnover from methodological differences among public genomes.

## 5. Conclusions

The mitochondrial reference of *C. ficifolia* PB_CK648 (GenBank PZ823090) is a 784,544-bp, 43.09%-GC sequence with a typical cucurbit coding complement, abundant short repeats, and extensive plastid homology. The single-segment Oatk graph, 547.26× mean read depth, complete ≥100× coverage, and 366 HiFi reads spanning the terminal-to-start junction with ≥2-kb anchors provide strong sequence-level support for the submitted reference. These findings do not establish that one circular molecule is the unique or predominant mitochondrial configuration in vivo. At the largest 392-bp repeat, the native junctions were supported by 268 and 308 reads, whereas the reciprocal junctions were supported by one and zero reads, providing no robust evidence of reciprocal recombination. The other 258 ≥50-bp repeat pairs require equivalent validation. The 73 nonredundant mitochondrial–plastid homologous loci cover 9.53% of the reference. All 494 reported RNA-editing sites are bioinformatic predictions and are not presented as observed transcript-level events. Conserved mitochondrial genes place *C. ficifolia* as sister to the *C. pepo*–*C. maxima* pair, whereas whole-genome alignments show substantial turnover in homologous-block orientation and order.

## Figures and Tables

**Table 1:** Genome and gene-content features of the *Cucurbita ficifolia* PB_CK648 mitochondrial reference (GenBank PZ823090).

Feature	Value
GenBank accession	PZ823090
Assembly representation	One circular reference representation
Genome length	784,544 bp
GC content	43.09%
Ambiguous bases	0
Protein-coding genes	38 distinct genes; 42 loci
tRNA genes	26 distinct types; 37 loci
rRNA genes	3 genes; 3 loci
All annotated genes	67 distinct types; 82 loci
Complete codons analyzed	10,954
Bioinformatic RNA-editing predictions	494 predictions in 37 genes (Deepred-Mt posterior probability > 0.90)

Gene diversity and physical locus counts are reported separately.

**Table 2:** PacBio Revio HiFi sequencing and assembly-validation metrics for the *Cucurbita ficifolia* PB_CK648 mitochondrial reference (GenBank PZ823090).

Feature	Value
HiFi sequencing data	933,158 reads; 14.294 Gb; read N50 15,600 bp
Final Oatk graph	One 784,977-bp segment; one 433-bp self-link
Primary mitochondrial alignments	40,971 (MAPQ ≥ 20)
Read-back depth	Mean 547.26×; median 507×; range 240–3285×
Coverage breadth	100% at ≥100×
Terminal-to-start junction support	366 reads with ≥2-kb anchors; 159 reads with ≥5-kb anchors

## Data Availability

The *C. ficifolia* mitochondrial genome is available in GenBank under accession PZ823090. Raw PacBio Revio HiFi reads are deposited in the National Genomics Data Center (NGDC) under BioProject PRJCA072108; the associated BioSample and Run accession records are linked through that BioProject.

## Supplementary Materials

The following supporting information can be downloaded at: https://www.mdpi.com/article/10.3390/genes17091103/s1, Table S1. Codon counts and relative synonymous codon usage (RSCU) values for *Cucurbita ficifolia* mitochondrial protein-coding genes; Table S2. CDS extraction and codon-counting quality control for the *Cucurbita ficifolia* mitochondrial genome; Table S3. Repetitive-element summary for the *Cucurbita ficifolia* mitochondrial genome; Table S4. Summary of mitochondrial-plastid sequence homology in *Cucurbita ficifolia*; Table S5. High-probability bioinformatic C-to-U RNA-editing predictions summarized by mitochondrial protein-coding gene; Table S6. Mitochondrial accessions and conserved protein-coding genes used for phylogenomic analysis; Table S7. Filtered whole-mitogenome collinearity between adjacent Cucurbitaceae references; Table S8. Sequence resources, accessions, software, and analysis thresholds used in the study; and Table S9. PacBio Revio HiFi sequencing, assembly, and junction-validation metrics for the *Cucurbita ficifolia* PB_CK648 mitochondrial reference. File: Supplementary Tables S1–S9.xlsx. Additional assembly-validation materials are provided as Supplementary Figure S1. Graph- and HiFi-read-based validation of the submitted *Cucurbita ficifolia* PB_CK648 mitochondrial sequence representation; Supplementary Figure S2. PacBio HiFi read-depth profile across the *Cucurbita ficifolia* PB_CK648 mitochondrial genome; and Supplementary Figure S3. HiFi-read support for native and candidate recombinant-like junctions associated with the largest exact repeat in the *Cucurbita ficifolia* PB_CK648 mitochondrial assembly. File: Supplementary Figures.pdf.
