# Supplementary material for: The Cucurbita ficifolia Mitochondrial Genome: Repeat-Rich Architecture, Plastid Homology, and Synteny Turnover Across Cucurbitaceae
Source: Genes (Basel). 2026 Sep 11;17(9):1103. doi: 10.3390/genes17091103 (PMC13606409; doi:10.3390/genes17091103)
Supplement: Supplementary file 1 [file genes-17-01103-s001.zip › Supplementary Figures.pdf]

Supplementary Assembly-Validation Figures

The Cucurbita ficifolia Mitochondrial Genome: Repeat-Rich Architecture, Plastid Homology, and Synteny Turnover across Cucurbitaceae

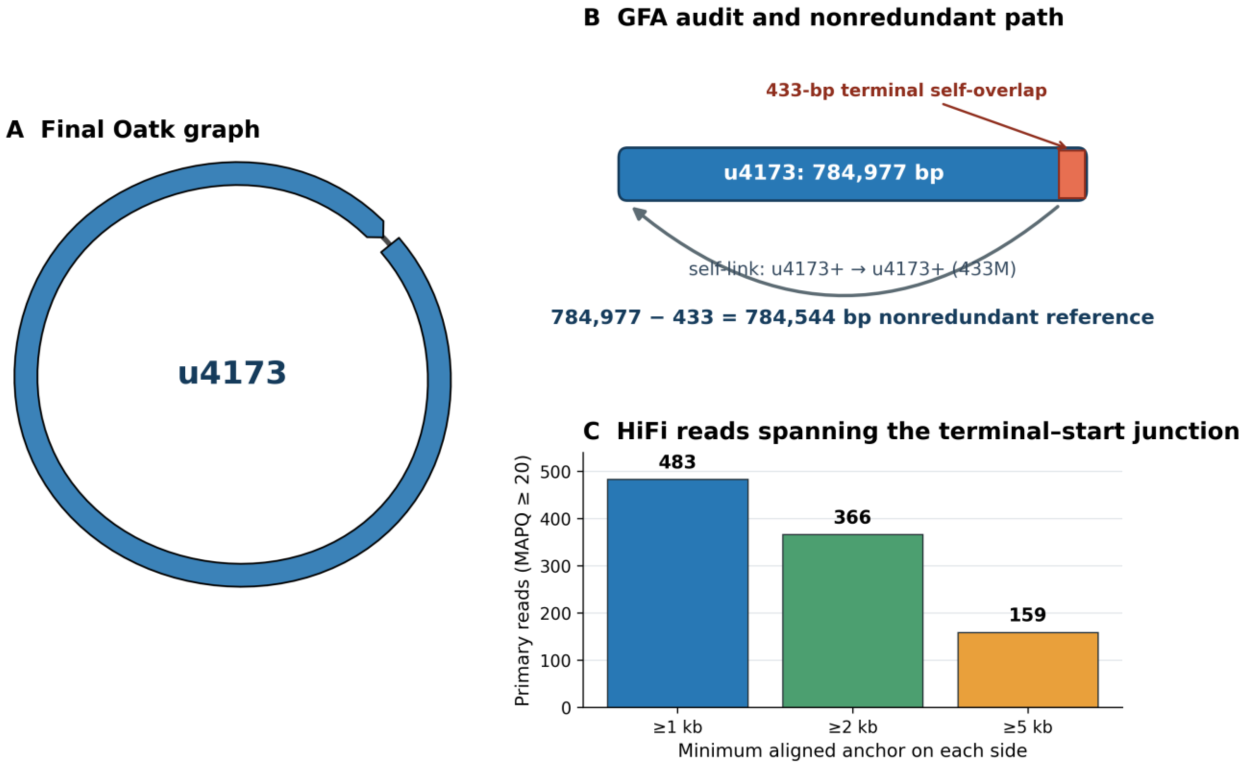

Supplementary Figure S1. Graph- and HiFi-read-based validation of the submitted *Cucurbita ficifolia* PB_CK648 mitochondrial sequence representation. (A) Bandage visualization of the final Oatk graph, which contains a single mitochondrial segment, u4173, connected to itself. (B) The 784,977-bp GFA segment carries a 433-bp terminal self-overlap, yielding a 784,544-bp nonredundant sequence identical in length to PZ823090. (C) Numbers of primary HiFi alignments (MAPQ ≥ 20) spanning the terminal-to-start junction with at least 1, 2, or 5 kb of aligned sequence on each side were 483, 366, and 159, respectively. These data support the terminal-to-start adjacency used in the submitted circular representation but do not establish that a 784,544-bp master circle is the unique or predominant *in vivo* molecular conformation.

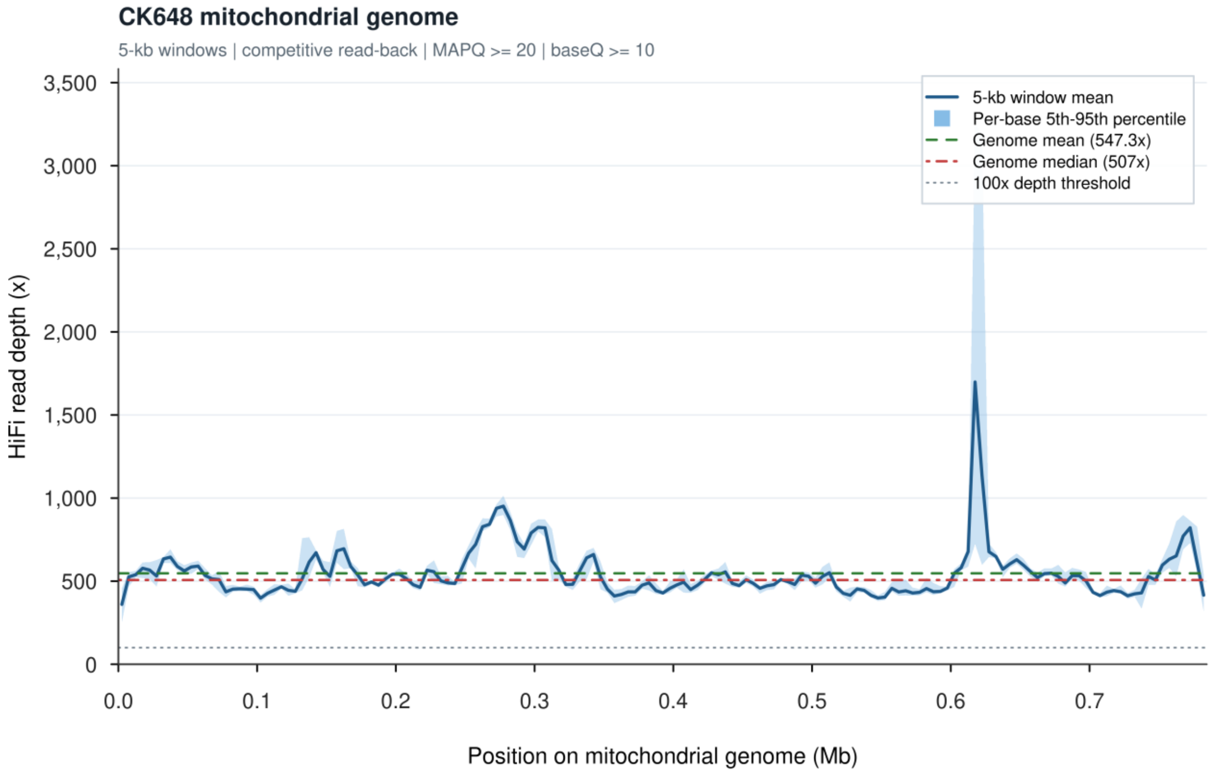

Supplementary Figure S2. PacBio HiFi read-depth profile across the *Cucurbita ficifolia* PB_CK648 mitochondrial genome. HiFi reads were competitively aligned against the mitochondrial and plastid references. The dark-blue line represents mean depth in nonoverlapping 5-kb windows, and the light-blue ribbon indicates the 5th–95th percentile of per-base depth within each window. Green and red dashed lines indicate genome-wide mean and median depth, respectively. Only primary alignments with MAPQ ≥ 20 were retained, and depth was calculated from bases with base quality ≥ 10. Across the 784,544-bp reference, mean and median depths were 547.26× and 507×, the range was 240–3,285×, and all positions were covered at ≥100×.

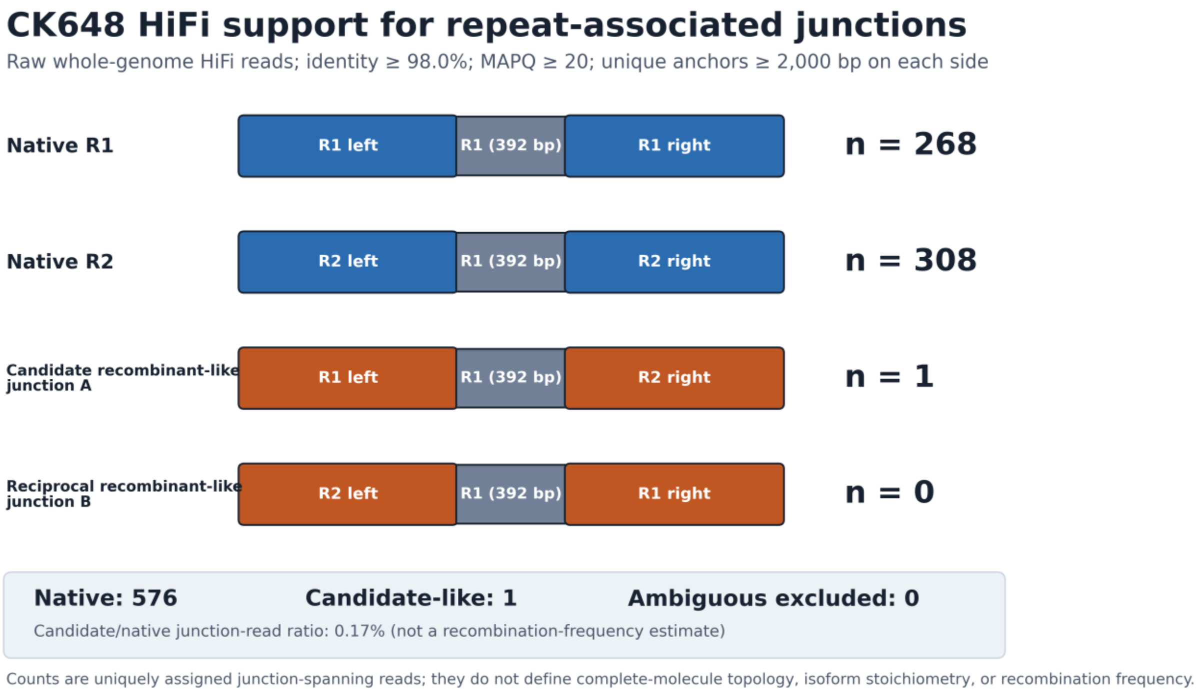

Supplementary Figure S3. HiFi-read support for native and candidate recombinant-like junctions associated with the largest exact repeat in the *Cucurbita ficifolia* PB_CK648 mitochondrial assembly. The 392-bp direct repeat R1 occurs at positions 92,091–92,482 and 680,968–681,359. Raw whole-genome HiFi reads were screened against four 12,392-bp references representing the two native and two reciprocal junctions. With identity ≥ 98%, MAPQ ≥ 20, and ≥2-kb unique anchors on each side, native R1 and R2 were supported by 268 and 308 unique reads, respectively. One read supported candidate recombinant-like junction A, whereas none supported reciprocal junction B. The isolated candidate read (MAPQ 60; 99.9832% identity; 6,000- and 5,501-bp anchors) is not considered conclusive evidence of robust reciprocal recombination, and the junction-read ratio should not be interpreted as molecular stoichiometry or recombination frequency.
